# Dihydroartemisinin Inhibits mTORC1 Signaling by Activating the AMPK Pathway in Rhabdomyosarcoma Tumor Cells

**DOI:** 10.3390/cells10061363

**Published:** 2021-06-01

**Authors:** Jun Luo, Yoshinobu Odaka, Zhu Huang, Bing Cheng, Wang Liu, Lin Li, Chaowei Shang, Chao Zhang, Yang Wu, Yan Luo, Shengyong Yang, Peter J. Houghton, Xiaofeng Guo, Shile Huang

**Affiliations:** 1 Department of Biochemistry and Molecular Biology, Louisiana State University Health Sciences Center, Shreveport, LA 71130-3932, USA; junluo@scau.edu.cn (J.L.); odakayu@ucmail.uc.edu (Y.O.); huangzhu@xmu.edu.cn (Z.H.); bing.cheng@lsuhs.edu (B.C.); wliu6@kumc.edu (W.L.); lin.li@lsuhs.edu (L.L.); chaowei.shang@lsuhs.edu (C.S.); zhangchao@ahmu.edu.cn (C.Z.); wuyang@scu.edu.cn (Y.W.); yan.luo@mayo.edu (Y.L.); 2 College of Veterinary Medicine, South China Agricultural University, Guangzhou 510642, China; 3 Research Center of Aquatic Organism Conservation and Water Ecosystem Restoration in Anhui Province, Anqing Normal University, Anqing 246011, China; 4 Key Laboratory of National Health and Family Planning Commission on Parasitic Disease Control and Prevention, Jiangsu Institute of Parasitic Diseases, Wuxi 214064, China; 5 Jiangsu Provincial Key Laboratory on Parasite and Vector Control Technology, Jiangsu Institute of Parasitic Diseases, Wuxi 214064, China; 6 State Key Laboratory of Biotherapy and Cancer Center, West China Hospital, Sichuan University, Chengdu 610041, China; yangsy@scu.edu.cn; 7 Greehey Children’s Cancer Research Institute, University of Texas Health Science Center, San Antonio, TX 78229-3000, USA; houghtonp@uthscsa.edu; 8 Department of Hematology and Oncology, Louisiana State University Health Sciences Center, Shreveport, LA 71130-3932, USA

**Keywords:** dihydroartemisinin, rhabdomyosarcoma, mTOR, AMPK, PTEN, raptor

## Abstract

Dihydroartemisinin (DHA), an anti-malarial drug, has been shown to possess potent anticancer activity, partly by inhibiting the mammalian target of rapamycin (mTOR) complex 1 (mTORC1) signaling. However, how DHA inhibits mTORC1 is still unknown. Here, using rhabdomyosarcoma (RMS) as a model, we found that DHA reduced cell proliferation and viability in RMS cells, but not those in normal cells, which was associated with inhibition of mTORC1. Mechanistically, DHA did not bind to mTOR or FK506 binding protein 12 (FKBP12). In addition, DHA neither inhibited insulin-like growth factor-1 receptor (IGF-1R), phosphoinositide 3-kinase (PI3K), and extracellular signal-regulated kinase ½ (Erk1/2), nor activated phosphatase and tensin homolog (PTEN) in the cells. Rather, DHA activated AMP-activated protein kinase (AMPK). Pharmacological inhibition of AMPK, ectopic expression dominant negative or kinase-dead AMPK, or knockdown of AMPKα attenuated the inhibitory effect of DHA on mTORC1 in the cells. Additionally, DHA was able to induce dissociation of regulatory-associated protein of mTOR (raptor) from mTOR and inhibit mTORC1 activity. Moreover, treatment with artesunate, a prodrug of DHA, dose-dependently inhibited tumor growth and concurrently activated AMPK and suppressed mTORC1 in RMS xenografts. The results indicated that DHA inhibits mTORC1 by activating AMPK in tumor cells. Our finding supports that DHA or artesunate has a great potential to be repositioned for treatment of RMS.

## 1. Introduction

Rhabdomyosarcoma (RMS) is the most common soft-tissue sarcoma, which often occurs in the head, neck, bladder, vagina, uterus, arms, legs, and trunk [1]. Approximately 80% of RMS patients are younger than 15 years old [1]. Histologically, RMS manifests in two major types, embryonal (ERMS) and alveolar (ARMS) [2]. Morphologically, the embryonic type resembles to the embryonic muscle cell precursor, whereas the alveolar type has clusters of round cells similar to lung alveoli [1,2]. Approximately 80% of ARMS tumors are characterized with the translocations or expression of the *PAX3/7*–*FOXO1* fusion transcript, resulting in overexpression of receptor tyrosine kinases such as fibroblast growth factor receptor 4 (FGFR4), hepatocyte growth factor receptor (HGFR, also named MET), and insulin-like growth factor 1 receptor (IGF-1R) [1,2]. In addition, insulin-like growth factor 2 (IGF-2) is upregulated by *PAX3-FOXO1* in RMS, activating the IGF-1R pathway [2]. Hence, the mammalian target of rapamycin (mTOR) pathway is frequently and constitutively activated in ARMS tumors, which have higher propensity for metastasis [1,2]. 

RMS is generally treated with surgery, radiation therapy, and chemotherapy [1,3]. The 5-year survival rate for children having low-to-intermediate-risk RMS ranges from 50% to 90%, while for high-risk patients (having metastatic or recurrent disease), the 5-year survival rate is less than 30% [1,2]. The standard chemotherapy regimen for RMS is the combination of vincristine, actinomycin D, and cyclophosphamide [1,3]. However, these chemotherapeutic treatments for children have long-term side effects, such as secondary cancers and infertility [1,3]. In recent clinical trials, targeted therapies and immunotherapies have shown improvements in the outcomes in patients with RMS, but the clinical benefit is still limited [4]. Therefore, there is a great need to develop novel systemic treatments, which have better efficacy with long-term safety, for RMS patients.

mTOR is a central controller for protein synthesis, cell growth, proliferation, and survival [5,6]. The dysregulation of the mTOR pathway correlates to tumor development and progression, so mTOR has become a hot target for cancer therapy [5,6]. mTOR functions as two complexes (mTORC1 and mTORC2) in mammalian cells [5,6]. mTORC1 consists of mTOR, mLST8 (mammalian lethal with sec-13 protein 8), raptor (regulatory-associated protein of mTOR), PRAS40 (proline-rich Akt substrate 40), and DEPTOR, whereas mTORC2 is composed of mTOR, mLST8, mSin1, rictor (rapamycin-insensitive companion of mTOR), mSin1 (mammalian stress-activated protein kinase-interacting protein 1), protor (protein observed with rictor), and DEPTOR [5,6]. Of note, raptor is essential for the assembly of mTORC1 and for recruiting mTOR substrates [7]. mTORC1 senses growth factors, nutrients (amino acids), energy, oxygen, and DNA damage, while mTORC2 primarily senses growth factors [5,6]. Both mTORC1 and mTORC2 can be positively regulated by the IGF-IR-phosphatidylinositol-3 kinase (PI3K), which is antagonized by phosphatase and tensin homolog (PTEN) [5,6]. mTORC1 can also be positively regulated by the Ras-Raf-MEK-Erk pathway [5,6]. In addition, mTORC1 is negatively regulated by AMP-activated protein kinase (AMPK) [8]. In response to low energy levels, AMPK is activated, which can phosphorylate tuberous sclerosis complex 2 (TSC2) at multiple sites (including S1387), promoting the formation and activation of TSCs [8], which antagonizes Rheb (Ras homolog enriched in the brain) by hydrolyzing GTP-Rheb to GDP-Rheb, thereby inhibiting Rheb-mediated mTORC1 [5,6]. Besides, activated AMPK can also phosphorylate raptor (S792), resulting in the inhibition of mTORC1 [9]. While p70 S6 kinase 1 (S6K1) and eukaryotic initiation factor 4E (eIF4E) binding protein 1 (4E-BP1) are two well-known substrates of mTORC1, Akt (S473) is the best characterized substrate of mTORC2 [5,6].

Rapamycin and its analogs (e.g., temsirolimus and everolimus) (termed rapalogs) were developed as the first generation of mTOR inhibitors [5,6]. Mechanistically, rapalogs do not impair mTOR’s kinase activity per se but first form a complex with the FK506 binding protein 12 (FKBP12) and then bind the FKBP12-rapamycin-binding (FRB) domain of mTOR, disrupting mTORC1 assembly and thus inhibiting mTORC1 [5,6]. However, rapalogs alone lack efficacy in treating most types of cancer, including RMS [5,6,10,11]. This is possibly due to the fact that the phosphorylation of 4E-BP1 (cap-dependent translation) is largely insensitive to rapalogs, and Akt (pro-survival) can be activated by rapalogs via a negative feedback mechanism [5,12,13,14]. Recently, mTOR kinase inhibitors (e.g., AZD8055 and INK128), called the second generation of mTOR inhibitors, have emerged, which compete with ATP in the catalytic site of mTOR and inhibit both mTORC1 and mTORC2 [5,6]. However, prolonged treatment with these inhibitors can also result in re-activation of Akt [15], highlighting resistance as a key problem that must be tackled by the new generation of mTOR inhibitors [5].

Dihydroartemisinin (DHA) is a derivative of artemisinin originally isolated from the plant *Artemisia annua* [16]. DHA is also the active metabolite of artemisinins, such as artemisinin, artesunate, and artemether [16,17]. Artemisinins have been widely used to treat malaria in children and adults showing high efficacy and safety [16,17,18,19]. Increasing evidence has demonstrated that artemisinins also possess potent anticancer effects on diverse tumor cell lines [19]. Artesunate, a water-soluble artemisinin derivative, has been in clinical trials for treatments of lung, cervical, breast, and colon cancers [19]. Multiple anticancer action modes of artemisinins have been described, including the induction of cell cycle arrest, apoptosis, autophagy, as well as the inhibition of cell invasion/motility and angiogenesis [19]. Correspondingly, DHA has been shown to alter the expression/activity of a variety of signaling molecules, such as MYC, cyclin-dependent kinases (CDKs), vascular endothelial growth factor receptor (VEGF), focal adhesion kinase (FAK), and hypoxia-inducible factor 1-alpha (HIF-1α) [19]. Recently, we and others have demonstrated that DHA inhibits mTOR [20,21,22,23,24,25,26,27,28,29,30,31,32,33,34]. Since many of those molecules (e.g., MYC, CDKs, VEGF, FAK, and HIF-1α) targeted by DHA are also directly or indirectly regulated by mTORC1 [5,6], we proposed that mTORC1 may be a major target of DHA for its anticancer activity, and DHA is a new inhibitor of mTORC1.

To the best of our knowledge, no study has determined how DHA inhibits mTORC1. In this study, we evaluated the anticancer activity of DHA in RMS cells in cell culture and in xenografts in mice. Using RMS as a model, we focused on determining the molecular mechanism by which DHA inhibits mTORC1 in tumor cells.

## 2. Materials and Methods

### 2.1. Materials

DHA (purity: >98% by HPLC; TCI America, Portland, OR, USA) was dissolved in dimethyl sulfoxide (DMSO) to prepare a stock solution (10 mM), aliquoted and stored at −20 °C. [10−*^3^*H]-dihydroartemisinin (specificity activity: 2.5 Ci/mmol; concentration: 1.0 mCi/mL in a hexane:ethanol (*v*:*v*, 7:3) solution; radiochemical purity: 98.5%) was obtained from Moravek Biochemical (Brea, CA, USA). Compound C (EMD Millipore, Burlington, MA, USA) was dissolved in DMSO to prepare a 10 mM stock solution and stored at −20 °C. RPMI 1640, Dulbecco’s modified Eagle’s medium (DMEM) (high glucose), DMEM/F12, 0.05% trypsin-EDTA, and Matrigel membrane matrix were obtained from Corning (Corning, NY, USA), and fetal bovine serum (FBS) was from R&D Systems (Minneapolis, MN, USA). For Western blotting or immunoprecipitation, the following antibodies were used: Erk2, c-Jun, p-c-Jun (Ser63), HIF-1α, REDD1, IGF-1Rβ, p-IGF-1Rβ (Tyr1161), mTOR, S6K1, S6, PI3K, Akt, β-actin, c-Myc, GAPDH (Santa Cruz Biotechnology, Dallas, TX, USA), p-Erk1/2 (Thr202/Tyr204), p38, p-p38 (Thr180/Tyr182), p-S6K1 (Thr389), p-AMPKα (Thr172), AMPKα, p-ACC (Ser79), ACC, p-S6 (Ser235/236), 4E-BP1, p-4E-BP1 (Thr37/46), p-4E-BP1 (Thr70), PDK1, p-PDK1 (Ser241), PTEN, p-PTEN (Ser380/Thr382/383), p-PI3K p85 (Tyr458)/p55 (Tyr199), p-Akt (Ser473), mLST8 (GβL) (Cell Signaling Technology, Danvers, MA, USA), raptor, rictor (Bethyl Laboratories, Montgomery, TX, USA), β-tubulin (Sigma-Aldrich, St. Louis, MO, USA), goat anti-mouse IgG-horseradish peroxidase, and goat anti-rabbit IgG-horseradish peroxidase (Pierce, Rockford, IL, USA). All other chemicals were obtained from Sigma-Aldrich (St. Louis, MO, USA) unless specified elsewhere.

### 2.2. Cell Lines and Culture 

Human RMS (Rh30, RD, Rh18, Rh28, Rh36, and Rh41) and Ewing sarcoma cells (Rh1, also named EW8) which were gifts from Dr. Peter J. Houghton, University of Texas Health Science Center, San Antonio, TX, USA were grown in RMPI 1640 supplemented with 10% FBS. Human primary skeletal muscle cells (#PCS-950-010, American Type Culture Collection (ATCC), Manassas, VA, USA) were cultured in a Mesenchymal Stem Cell Basal Medium (#PCS-500-030, ATCC) supplemented with Primary Skeletal Muscle Cell Growth Kit (PCS-950-040, ATCC), while human dermal primary fibroblasts (#PCS-201-012, ATCC) were grown in a Fibroblast Basal Medium supplemented with Fibroblast Growth Kit-Low Serum (#PCS-201-041, ATCC); both of them were used within 6 passages. Mouse muscle myoblasts (C2C12 and ATCC), raptor, rictor-inducible knockout (KO) mouse embryonic fibroblasts (MEFs, SV40 large T-antigen-immortalized and expressing the Cre/LoxP system) (gifts from Dr. Michael Hall, University of Basel, Switzerland), and 293A cells (Invitrogen, Calsbad, CA, USA) were cultured in DMEM supplemented with 10% FBS. To induce the KO of raptor or rictor, the MEFs were treated with 1 μM 4-hydroxytamoxifen (Sigma-Aldrich) for 3 days [35]. All cell lines were cultured in a humid incubator (37 °C and 5% CO_2_) and trypsinized with a 0.05% trypsin–EDTA solution for subculture or experiments.

### 2.3. Cell Proliferation and Viability Assays

Cell proliferation and viability were evaluated by cell counting and MTS assay, as described previously [36]. Treatment with DMSO (vehicle) served as a control.

### 2.4. [^3^H]-DHA Labeling In Vivo

Rh30 cells were seeded in 100 mm culture dishes (3 × 10^6^ cells/dish) for culture. The next day, the cells were labeled with 10 μCi [^3^H]-DHA for 21 h. Subsequently, the cells were briefly washed with PBS and lysed in an ice-cold CHAPS lysis buffer (40 mM HEPES, pH 7.4, 120 mM NaCl, 1 mM EDTA, 10 mM pyrophosphate, 10 mM glycerophosphate, 50 mM NaF, 1.5 mM Na_3_VO_4_, 0.3% (*w*/*v*) CHAPS, and a cocktail of protease inhibitors (dilution, 1:1000; Sigma-Aldrich). The cell lysates were sonicated for 20 s and centrifuged at 13,000 rpm and at 4 °C for 3 min. The supernatants were transferred to fresh Eppendorf tubes. The protein concentration in the supernatants was determined using a BCA kit (Pierce). Supernatants with an equal amount (700 μg) of crude protein were incubated with 30 μL of protein A/G agarose beads and 3 μg of antibodies to goat anti-mTOR antibody or normal goat IgG on a rotator overnight at 4 °C. The agarose beads were collected by centrifugation at 3500 rpm and at 4 °C for 3 min and washed once with 1 mL of an ice-cold CHAPS buffer and three additional washes with ice-cold PBS. The relative radioactivity (cpm) of immunoprecipitated products was measured on a Beckman LS6500 scintillation counter (Beckman Coulter, Fullerton, CA, USA).

### 2.5. Recombinant Adenoviruses, Lentiviral shRNAs, and Infection of Cells

Recombinant adenoviruses expressing green fluorescent protein (GFP) and MYC-tagged dominant negative (DN) AMPKα1 (D157A) (Ad-AMPK-DN) were described previously [37]. Recombinant adenovirus expressing MYC-tagged kinase-dead AMPKα2 (K45R) (Ad-AMPK-KD) [38] was a gift from Dr. Nicholas J. G. Webster (University of California, San Diego, CA). For experiments, the cells were infected with and individual adenovirus at a multiplicity of infection (MOI) of 5 for 24 h. Subsequently, the infected cells were used for experiments. Cells infected with Ad-GFP served as control. The expression of MYC-tagged AMPK-DN or AMPK-KD was determined by Western blotting with antibodies to c-Myc.

Lentiviral shRNAs to human raptor, rictor, AMPKα1, and GFP were described previously [39,40]. For use, monolayer cells, when grown to about 70% confluence, were infected with an individual lentivirus in the presence of 8 μg/mL polybrene for 12 h twice at an interval of 6 h. Uninfected cells were eliminated by exposure to 2 μg/mL puromycin for 48 h before use.

### 2.6. Western Blotting

Western blotting was performed as described [21].

### 2.7. Co-Immunoprecipitation of mTOR and In Vitro mTOR Kinase Assay

Rh30 cells were seeded in 100 mm culture dishes (3 × 10^6^ cells/dish) and grown overnight. The cells were then treated with DHA (0–30 μM) for 24 h. After aspirating the used medium, the cells were briefly washed with PBS and lysed in an ice-cold CHAPS lysis buffer, followed by immunoprecipitation with goat anti-mTOR antibody or normal goat IgG (as a control). Finally, to detect the interaction of mTOR with raptor, rictor, and mLST8, the immunoprecipitants were subjected to immunoblotting with antibodies to mTOR, raptor, rictor, and mLST8, as described [41]. To detect the mTORC1 activity, the above immunoprecipitants were utilized for the in vitro mTOR kinase assay, as described [41].

### 2.8. Molecular Docking

The molecular docking studies were performed using Genetic Optimization of Ligand Docking (GOLD) 5.0 and LibDock [42,43]. The 3-D structures of mTOR and the protein complex of FKBP12 and the FRB domain of mTOR were taken from the PDB database with the PDB entries being 4JT5 and 3FAP, respectively [44,45], while the 3-D structure of the protein complex of AMPK and A-769662 was taken from the PDB database (PDB entry: 4CFF) [46]. Discovery Studio 3.1 (Accelrys, San Diego, CA, USA) software package was used to prepare the protein structures including adding hydrogen atoms to the protein, removing water molecules, and assigning force fields (here the CHARMM force field was adopted). The binding site was defined as a sphere containing residues that remained within 9 Å (for mTOR) or 10 Å (for AMPK) of the ligand, an area large enough to cover the ligand-binding region at the domain of proteins. The binding affinity was estimated using LibDock score and/or GOLD score.

### 2.9. Study in Rhabdomyosarcoma Xenografts

To study the inhibitory effect of DHA on tumor growth in vivo, artesunate (ART), a pro-drug of DHA, was used. CB17SC scid^−/−^ female mice (Taconic Farms, Germantown, NY) were maintained under barrier conditions, and experiments were conducted using the protocols approved by the institutional animal care and use committee (ethical code number: LSUHSC-S #P20-003; the date of approval: 30 August 2019). Human Rh30 cells (6 × 10^6^ cells resuspended in 100 μL of a 1:1 (*v*:*v*) solution of serum-free DMEM/matrigel) were injected into the right flank of each mouse. Seven days later, the animals were randomized into 5 groups (10 mice/group). Then, the mice were intraperitoneally (i.p.) injected once daily with artesunate (dissolved in 5% Na_2_CO_3_ and diluted in 0.9% NaCl) at doses of 25, 50, 100, or 150 mg/kg body weight) or with vehicle control, as described [47]. Tumor volume (calculated as: length × width^2^/2) was determined with a digital caliper every 2–3 days. At the end of the experiment, all animals were sacrificed, and the tumors were collected and analyzed.

To study the in vivo effect of DHA on AMPK and mTORC1, female C.B.17SC scid^−/−^ mice (5–6 weeks old) bearing Rh65 xenografts were treated i.p. with DHA (100 mg/kg body weight). Following treatment for 2, 4, 8, and 24 h, the mice (3 mice per time point) were sacrificed, and the tumor tissues were collected, frozen in liquid nitrogen and stored at –80 °C for further analysis. Non-treatment with DHA served as a control. Tumor lysates were analyzed by Western blotting with indicated antibodies.

### 2.10. Statistical Analysis

All data were expressed as mean values ± SD. Data were analyzed using GraphPad Prism 6 software (GraphPad Software, La Jolla, CA, USA). Group variability and interaction were compared using Student’s *t*-test or one-way ANOVA followed by Bonferroni’s post-tests to compare replicate means. A level of *p* < 0.05 was considered to be statistically significant.

## 3. Results

### 3.1. DHA Inhibits Cell Proliferation and mTORC1 Signaling in RMS Cells

**Figure 1:**
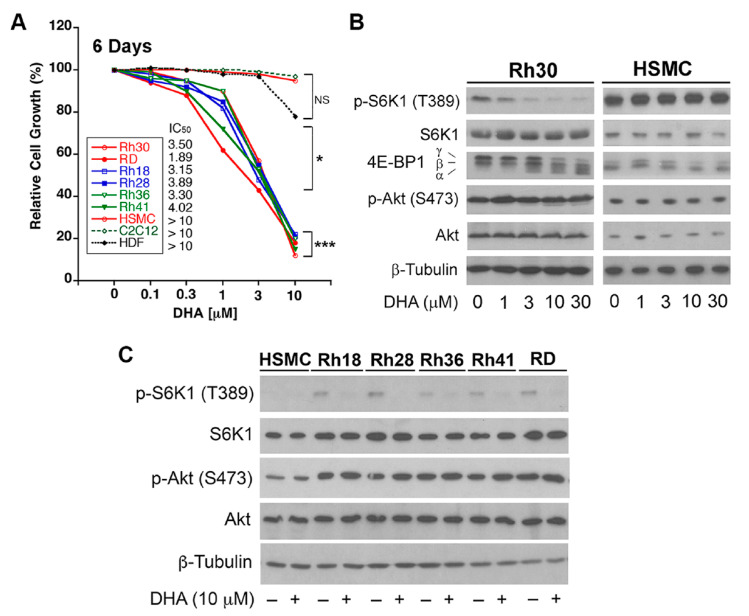
Dihydroartemisinin (DHA) inhibits the proliferation of rhabdomyosarcoma (RMS) cells. (**A**) Indicated cell lines were exposed to DHA (0−10 µM) for 6 days, followed by cell counting using a Beckmann Coulter counter. The results are shown as mean values (*n* = 3). NS, not significant; * *p* < 0.05; *** *p* < 0.001; difference versus vehicle control group. (**B**,**C**) Indicated cells were treated with DHA at indicated concentrations for 24 h, followed by Western blotting with indicated antibodies.

To reposition DHA for treatment of RMS, six RMS cell lines (Rh30, RD, Rh18, Rh28, Rh36, and Rh41) were employed for the growth inhibition assay. As RMS develops primarily from skeletal muscle cells, normal human primary skeletal muscle cells (HSMCs) and mouse skeletal muscle cells (C2C12) were used as normal controls. As shown in Figure 1A, RMS cell lines tested were sensitive to DHA, with the half maximal inhibitory concentrations (IC_50_) = 1.89−4.02 µM. In contrast, normal skeletal muscle cells (HSMCs and C2C12) were resistant to DHA (IC_50_ > 10 µM). Similar results were observed in normal human primary dermal fibroblasts (HDFs). The results suggested that DHA, at pharmacological concentrations (<10 µM), has little effects on normal cell growth and can selectively target RMS tumor cells.

mTOR is a central controller of cell growth, proliferation, and survival [5,6]. Next, we wondered whether DHA reduction of cell proliferation is related to the inhibition of mTOR. For this, Rh30 cells and HSMCs were treated with DHA (0–30 µM) for 24 h, followed by Western blotting. In line with the above growth inhibitory effect (Figure 1A), the treatment with DHA inhibited mTORC1-mediated phosphorylation of S6K1 and 4E-BP1 in a concentration-dependent manner in RMS (Rh30) cells, but not in normal cells (HMSCs) (Figure 1B). Consistent with our previous findings in Rh1 and C2C12 cells [21], DHA treatment did not impact mTORC2-mediated phosphorylation of Akt (S473) in both Rh30 cells and HSMCs (Figure 1B). Similar results were observed in other RMS cells (Rh18, Rh28, Rh36, Rh41, and RD) (Figure 1C). Of note, the basal phosphorylation levels of S6K1 and Akt were much higher in RMS cells than in normal cells (HSMCs) (Figure 1C), suggesting that the mTOR signaling is hyperactive in RMS cells. The results highlight that DHA is a novel inhibitor of mTORC1.

**Figure 2:**
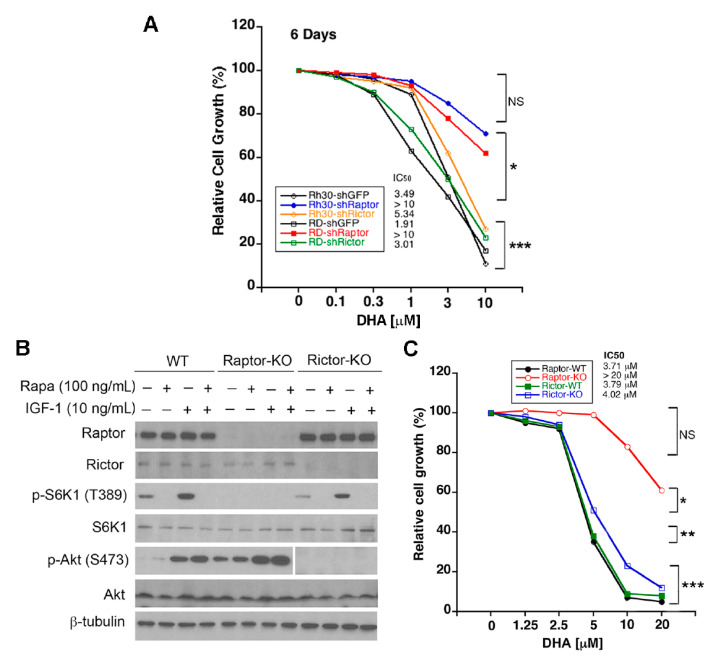
Disruption of mammalian target of rapamycin complex 1 (mTORC1), but not mammalian target of rapamycin complex 2 (mTORC2), confers high resistance to DHA-induced cell growth suppression. (**A**) Rh30 and RD cells, infected with lentiviral shRNAs to raptor, rictor, or GFP (control), were treated with DHA (0−10 µM) for 6 days, followed by cell counting using a Beckmann Coulter counter. (**B**) Raptor or rictor-inducible knockout mouse embryonic fibroblasts (MEFs) were treated with or without 4-hydroxytamoxifen (4-OHT) (1 µM) for 3 days, to generate raptor-WT, raptor-KO, rictor-WT, and rcitor-KO cells. Western blotting was performed with indicated antibodies. (**C**) Indicated cells, seeded in 96-well plates (all at 4 × 10^3^ cells/well), were treated with DHA (0–20 µM) for 72 h, followed by MTS assay. Shown are mean values (*n* = 3). NS, not significant; * *p* < 0.05; ** *p* < 0.01; *** *p* < 0.001, difference versus vehicle control group (**A**,**C**).

Raptor and rictor are essential for the activity of mTORC1 and mTORC2, respectively [5,6]. Loss of mTORC1 or mTORC2 function (KO of raptor or rictor) reduces the growth rates of cells [35]. If mTORC1 is the major target for DHA-mediated RMS cell growth suppression, depletion of raptor should confer resistance to DHA. To this end, lentiviral shRNAs to raptor, rictor, and GFP (control) were employed [40]. Consistent with our previous report [40], the infection of Rh30 cells with lentiviral shRNAs to raptor and rictor downregulated the protein levels of raptor and rictor by 90% and 85%, respectively, in the cells compared to in controls. Similar to KO of raptor or rictor in MEFs [35], the knockdown of raptor or rictor in RMS cells also inhibited cell proliferation (Supplementary Figure S1). Of interest, knockdown of raptor, but not rictor, rendered high resistance to DHA-induced cell growth inhibition in Rh30 and RD cells (Figure 2A). To validate the finding, SV40 large T-antigen-immortalized raptor and rictor-inducible KO MEFs [35] were utilized. As expected, the treatment with 1 μM 4-hydroxytamoxifen for 3 days resulted in the deficiency of raptor or rictor in corresponding MEFs (Figure 2B). The KO of raptor inhibited p-S6K1 (T389), while the KO of rictor inhibited p-Akt (S473) in the cells (Figure 2B), indicating the loss of mTORC1 and mTORC2 in these MEFs, respectively. Following 72 h treatment with DHA, raptor-WT (wild-type), rictor-WT, and rictor-KO MEFs were sensitive to DHA (IC_50_ = 3.71–4.02 µM), whereas raptor KO MEFs were highly resistant to DHA (IC_50_ > 20 µM) (Figure 2C). Collectively, these observations support our hypothesis that DHA may execute its anticancer action primarily by targeting mTORC1 signaling.

### 3.2. DHA Does Not Bind to mTOR or FKBP12

**Figure 3:**
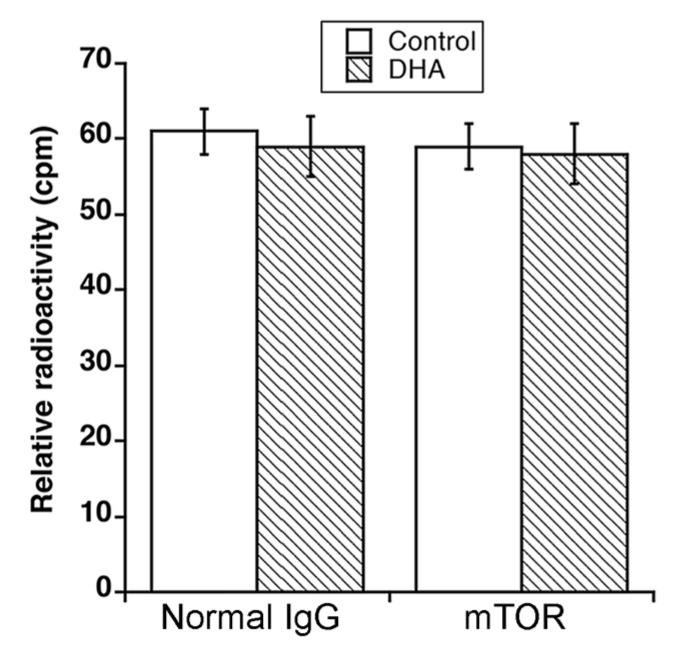
DHA does not directly bind to mTOR or FKBP12. Rh30 cells were pretreated with in vivo labeled with 10 μCi [^3^H]-DHA for 21 h. The cell lysates were used for immunoprecipitation with anti-mTOR antibodies or normal IgG (control). Shown is the relative radioactivity (cpm) of immunoprecipitation products.

To determine how DHA inhibits mTORC1 signaling, first of all, we investigated whether DHA binds to mTOR. For this, Rh30 cells were labeled with 10 µCi [^3^H]-DHA for 21 h, followed by immunoprecipitation with mTOR antibodies or normal IgG (control). No significant amount of [^3^H]-DHA was detected in the immunoprecipitates of mTOR, compared to in normal IgG (Figure 3), suggesting that DHA does not bind to mTOR directly.

**Figure 4:**
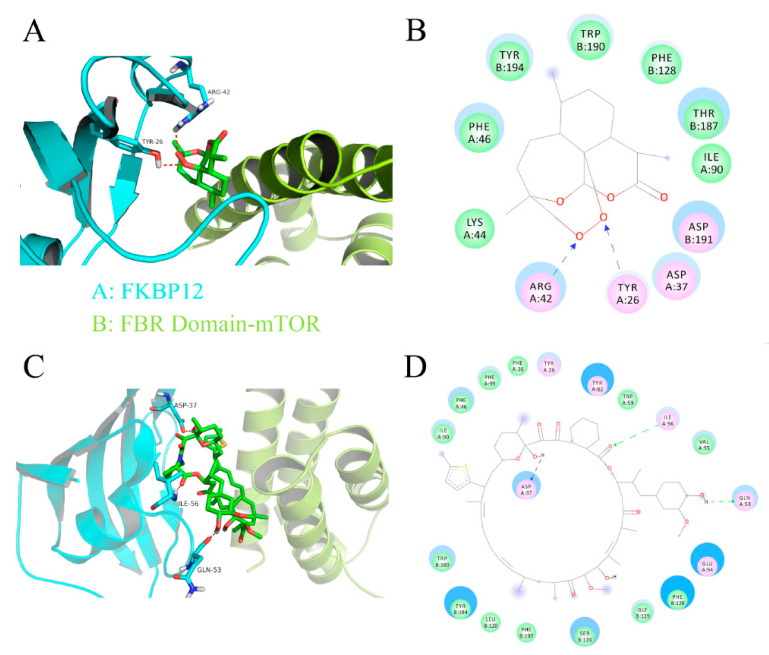
Predicted binding modes of dihydroartemisinin (**A**,**B**) and rapamycin (**C**,**D**) in the interface cavity of FKBP12 and the FRB domain of mTOR. The 3-D structure of the protein complex of FKBP12 and the FRB domain was taken from the PDB database (PDB ID: 3FAP). The calculated binding mode of DHA in the interface cavity of FKBP12 and the FRB domain of mTOR is shown in (**A**,**B**). DHA, namely (3R,12aR)-octahydro-12H-3,12-epoxy[1,2]dioxepino[4,3-i] isochromen-10(3H)-one, is sandwiched between FKBP12 and the FRB domain of mTOR. Two hydrogen bonds are formed between oxygen atoms of DHA and residues ARG42 and TYR26 of FKBP12. The seven-membered ring and the two six-membered rings in DHA form hydrophobic interactions with residues LYS44, PHE46, TYR194, TRP190, PHE128, THR187, and ILE90 in the protein complex of FKBP12 and the FRB domain of mTOR. For comparison, the binding mode of rapamycin in the interface cavity of FKBP12 and FRB domain of mTOR (**C**,**D**). Obviously, rapamycin forms a much better interaction with FKBP12 than DHA does. Three hydrogen bonds are formed between rapamycin and FKBP12: the first one corresponds to that formed between the carbonyl group of rapamycin and the ILE56 residue in FKBP12, and the other two are between two hydroxyl groups of rapamycin and residues ASP37 and GLN53, respectively. Rapamycin also forms good hydrophobic interactions with residues TRP190, TYR194, LEU120, PHE197, SER124, GLY129, PHE128, TYR82, LEU56, VAL55, TRP59, PHE39, TYR26, PHE46, and Phe99 in mTOR. Collectively, although DHA has the possibility to bind the interface cavity of FKBP12 and the FRB domain of mTOR, the interaction of DHA with the protein complex is much weaker than that of rapamycin, consistent with the calculated scoring function values (see Table 1).

It is known that rapamycin firstly forms a complex with FKBP12 and then binds to the FRB domain of mTOR, inhibiting mTORC1 [5,6]. Next, we wondered whether DHA, such as rapamycin, inhibits mTORC1 by forming a complex with FKBP12. For this, molecular docking was performed. We found that DHA had a possibility to bind the interface cavity of FKBP12 and the FRB domain of mTOR, but the interaction of DHA with the protein complex was much weaker than that of rapamycin (Figure 4A–D), consistent with the calculated scoring function values (e.g., LibDock score: 29.72 for DHA vs. 101.40 for rapamycin) (Table 1). Collectively, our results indicated that DHA does not bind to mTOR or FKBP12 directly, suggesting that DHA inhibits mTORC1 through indirect mechanism(s).

### 3.3. DHA Does Not Alter the Phosphorylation of IGF-1R/PI3K/PTEN and Erk1/2 

**Figure 5:**
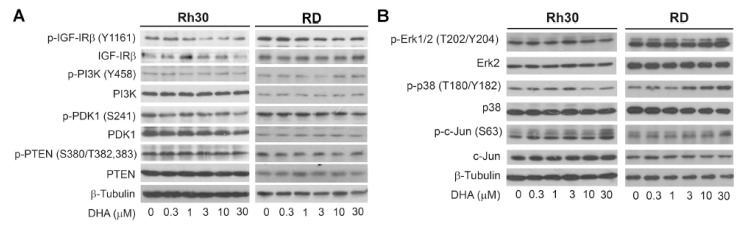
DHA does not alter the phosphorylation of IGF-1R/PI3K/PTEN and Erk1/2 in tumor cells. (**A**,**B**) Rh30 or RD cells were treated with DHA (0−30 μM) for 24 h, followed by Western blotting with indicated antibodies.

Since mTORC1 is positively regulated by the IGF-1R-PI3K-Akt and Ras-Raf-MEK-Erk pathways but negatively regulated by PTEN [5,6], we further tested whether DHA inhibits mTORC1 signaling indirectly by altering these upstream regulators in cells. The treatment with DHA (0–30 μM) for 24 h did not obviously alter the phosphorylation of IGF-1Rβ (Tyr1161), PI3K p85 (Tyr458), PDK1 (Ser241), and PTEN (Ser380/Thr382), as well as total cellular levels of these proteins (Figure 5A) and Akt (Figure 1B). Similarly, DHA did not apparently affect the phosphorylation or total protein level of Erk1/2 in the RMS cells (Figure 5B). Of note, at high concentrations (10–30 μM), DHA slightly inhibited the phosphorylation of p38 MAPK (p-p38) in Rh30 cells but moderately activated p-p38 in RD cells (Figure 5B). In addition, DHA (30 μM) induced the phosphorylation of c-Jun (a substrate of JNK) in both Rh30 and RD cells (Figure 5B). Hence, these data imply that DHA inhibits mTORC1, not by altering the phosphorylation of IGF-1R/PI3K/Akt, PTEN, and Erk1/2.

### 3.4. DHA Does Not Induce HIF-1α/REDD1 Expression, but Triggers AMPK Phosphorylation 

**Figure 6:**
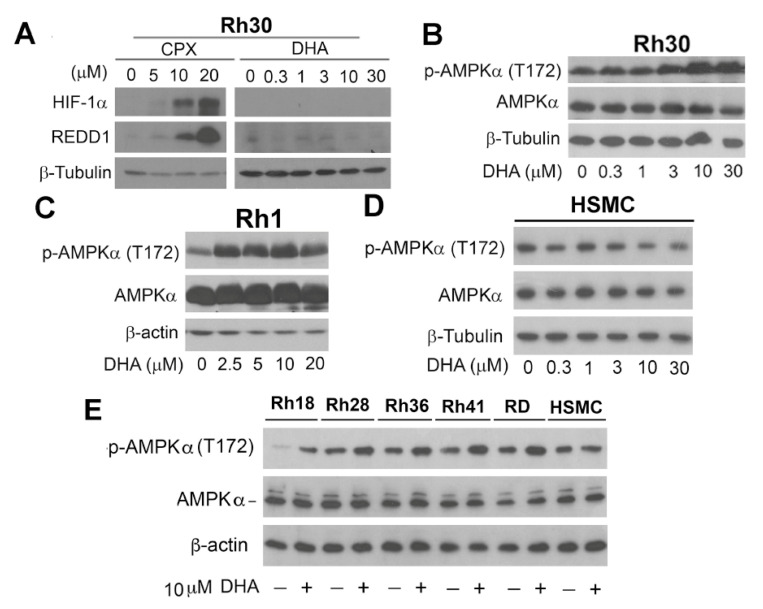
DHA does not induce HIF-1α/REDD1 expression, but triggers AMPK phosphorylation. (**A**) Rh30 cells were treated with DHA (0–30 μM) for 24 h, followed by Western blotting with indicated antibodies. Ciclopirox olamine (CPX) served as a positive control for the induction of HIF1α and REDD1. (**B**–**E**) Indicated cell lines were treated with DHA at indicated concentrations for 24 h, followed by Western blotting with indicated antibodies.

As the HIF1-REDD1 and AMPK pathways negatively regulate mTORC1 [8,9,48,49], next, we asked whether DHA inhibits mTORC1 signaling by activating these two pathways. To this end, tumor cells were treated with DHA (0–30 μM) for 24 h, followed by Western blotting. The results showed that the treatment with DHA did not induce the expression of HIF-1α or REDD1 (regulated in development and DNA damage responses 1) in Rh30 cells (Figure 6A). As a positive control [37], the treatment with ciclopirox olamine (CPX) induced the robust expression of HIF-1α and REDD1. Interestingly, DHA treatment induced the phosphorylation of the catalytic subunit of AMPK (p-AMPKα and T172) in Rh30 cells in a dose-dependent manner (Figure 6B). Similar results were also observed in Rh1, Rh18, Rh28, Rh36, Rh41, and RD cells (Figure 6C,E). Since DHA did not inhibit mTORC1 in normal HSMCs (Figure 1B), we also tested whether DHA affects p-AMPKα (T172) in this cell line. As shown in Figure 6D,E, treatment with DHA (0−30 μM) for 24 h had no evident impact on p-AMPKα in HSMCs. The results suggest that DHA inhibits mTORC1 possibly by activating AMPK.

### 3.5. DHA-Induced Activation of AMPK Contributes to the Inhibition of mTORC1

**Figure 7:**
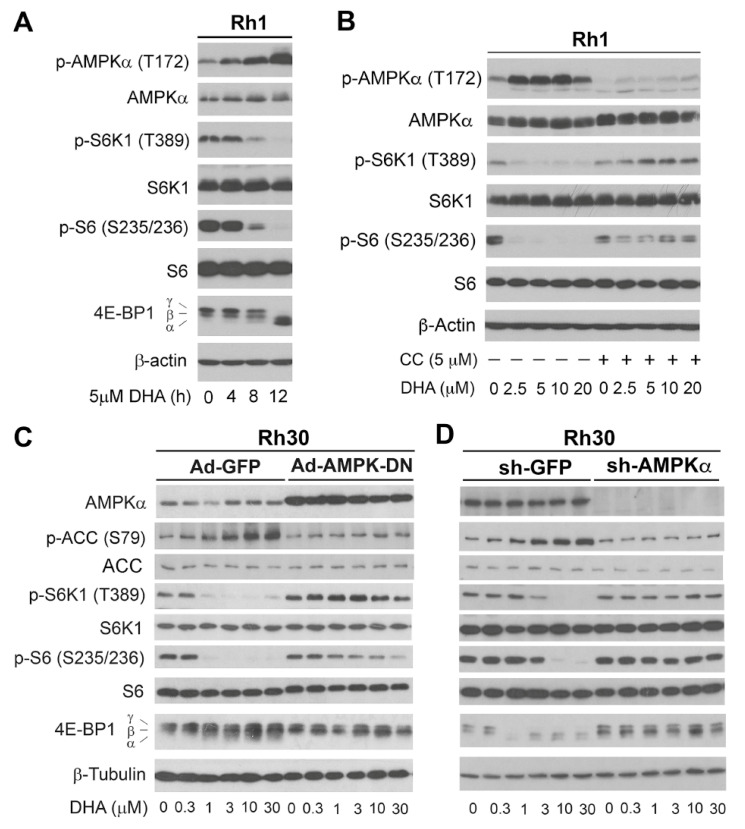
DHA-induced activation of AMPK contributes to inhibition of mTORC1. (**A**) Rh1 cells were treated with DHA (5 μM) for indicated time, followed by Western blotting with indicated antibodies. (**B**) Rh1 cells were pretreated with or without Compound C (CC) (5 µM) for 2 h, and then exposed to with or without DHA (5 µM) for 24 h, followed by Western blotting using indicated antibodies. (**C**) Rh30 cells were infected with recombinant adenovirus expressing myc-tagged dominant negative (DN) AMPK (Ad-AMPK-DN) or GFP (Ad-GFP) for 24 h, and then treated with DHA (0−30 μM) for another 24 h, followed by Western blotting with indicated antibodies. (**D**) Rh30 cells, infected with lentiviral shRNA to human AMPKα1 or GFP, were treated with DHA (0−30 μM) for 24 h, followed by Western blotting with indicated antibodies.

To investigate the relationship between the DHA-induced inhibition of mTORC1 and the activation of AMPK, a time-course experiment was performed. When Rh1 cells were treated with DHA (5 μM) for 0–12 h, the phosphorylation level of AMPKα increased in a time-dependent manner. The phosphorylation of AMPKα was modestly induced at 8 h and robustly induced at 12 h, which matched well with the inhibition pattern on mTORC1 (Figure 7A). The results suggest that DHA-induced mTORC1 inhibition may be associated with activation of AMPK.

To validate whether DHA-induced activation of AMPK contributes to inhibition of mTORC1, Rh1 cells were pre-treated with or without compound C (a selective inhibitor of AMPK) for 2 h and then exposed to DHA (5 μM) for 24 h. As predicted, pretreatment with compound C remarkably attenuated DHA-induced p-AMPKα (Figure 7B). Of interest, the inhibition of AMPK profoundly prevented DHA from inhibiting the phosphorylation of S6K1 and S6 (Figure 7B). Similar results were observed in Rh30 cells (Supplementary Figure S2). These data support that AMPK activation is involved in DHA-induced mTORC1 inhibition.

To corroborate the above finding, Rh30 cells were infected with a recombinant adenovirus expressing DN AMPKα (Ad-AMPK-DN), kinase-dead AMPKα (Ad-AMPK-KD) [37,38], or GFP (Ad-GFP, control) for 24 h, and then treated with DHA for another 24 h. As anticipated, ectopic expression of AMPK-DN or AMPK-KD, but not GFP, attenuated DHA-induced phosphorylation of ACC (S79), a substrate of AMPK (Figure 7C; Supplementary Figure S3), suggesting that both Ad-AMPK-DN and Ad-AMPK-KD were working well in the cells. Importantly, expression of AMPK-DN or AMPK-KD did render high resistance to the inhibitory effect of DHA on mTORC1 in the cells (Figure 7C; Supplementary Figure S3). Furthermore, similar results were observed in Rh30 cells when AMPKα1 was knocked down with lentiviral shRNA to AMPKα1 (Figure 7D). Together, our results indicate that activation of AMPK plays a critical role in DHA-induced inhibition of mTORC1.

### 3.6. DHA Dissociates Raptor from mTOR and Inhibits mTORC1 Activity

**Figure 8:**
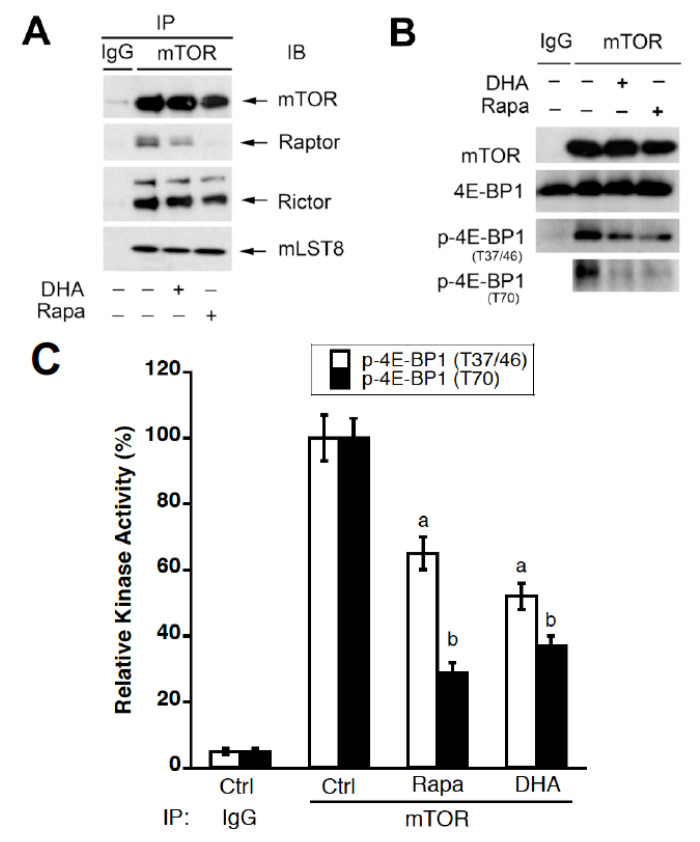
DHA dissociates raptor from mTOR and inhibits mTORC1 activity. (**A**,**B**) Rh30 cells were treated with or without DHA (3 µM) for 24 h or with rapamycin (Rapa, 100 ng/mL, positive control) for 2 h, followed by immunoprecipitation (IP) using antibodies to mTOR or normal IgG (control). The immunoprecipitates were then subjected to immunoblotting (IB) with indicated antibodies or were used for the in vitro mTOR kinase assay by incubating with recombinant 4E-BP1 protein (as a substrate) at room temperature for 30 min, followed by Western blotting with indicated antibodies. (**C**) Semi-quantitative data (mean ± SD) of three independent experiments for (**B**). ^a^ indicates the difference with the control group of p-4E-BP1 (T37/46) (*p* < 0.05); ^b^ indicates the difference with the control group of p-4E-BP1 (T70) (*p* < 0.05).

Rapamycin inhibits mTORC1 through the rapamycin-FKBP12 binding to mTOR, which results in the dissociation of raptor from mTOR, thereby inhibiting the mTORC1 function [7]. Having observed that DHA did not bind to mTOR (Figure 3), we further investigated whether DHA disrupts mTORC1. For this, Rh30 cells were treated with or without DHA (3 µM) for 24 h, or rapamycin (100 ng/mL, positive control) for 2 h, followed by immunoprecipitation with antibodies to mTOR or normal IgG (negative control). By immunoblotting, as expected, rapamycin did not obviously affect the binding of mTOR to mLST8 or rictor but dramatically reduced the interaction of mTOR with raptor (Figure 8A). Interestingly, DHA acted in the same way, although 3 µM of DHA did not cause the dissociation of raptor from mTOR so potently as 100 ng/mL of rapamycin (Figure 8A). The effect of DHA on the mTOR–raptor complex (mTORC1), but not the mTOR–rictor complex (mTORC2), was in line with our finding that DHA inhibits mTORC1-mediated phosphorylation of S6K1 and 4E-BP1 but does not affect mTORC2-mediated phosphorylation of Akt (Figure 1B).

As raptor is essential for the mTORC1 function [5,6], we further assessed the effect of DHA on the mTORC1 activity by in vitro mTOR kinase assay using recombinant 4E-BP1 protein as a substrate. As expected, the treatment with rapamycin (100 ng/mL) for 2 h strongly inhibited the mTORC1 activity in Rh30 cells, as the phosphorylation of 4E-BP1 on T37/46 and T70 was inhibited by approximately 40% and 70%, respectively (Figure 8B,C). Of interest, the treatment with DHA (3 µM) for 24 h inhibited the mTORC1 activity as potently as rapamycin (Figure 8B,C).

### 3.7. Artesunate Inhibits Tumor Growth, Suppresses mTORC1 and Activates AMPK in RMS Xenografts

**Figure 9:**
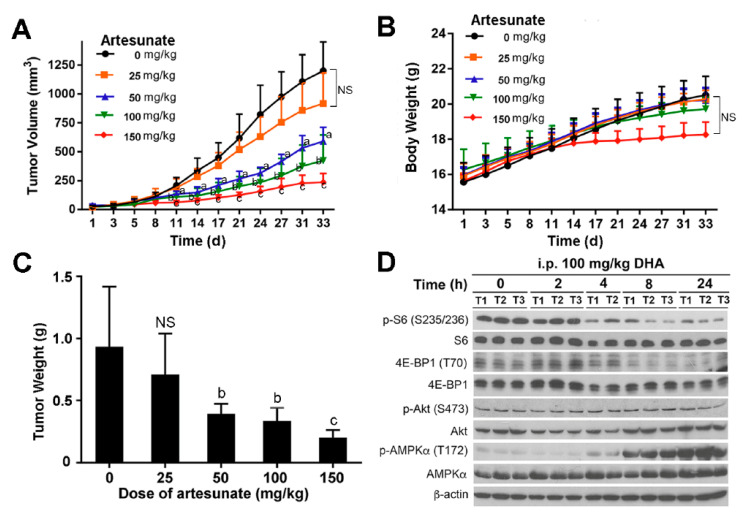
Artesunate inhibits tumor growth, suppresses mTORC1 and activates AMPK in RMS xenografts. (**A**–**C**) C.B.17SC scid^−/−^ female mice (5–6 weeks old) bearing Rh30 xenografts were treated intraperitoneally (i.p.) with artesunate at the indicated doses or vehicle (0.9% NaCl) once daily. Tumor volume (**A**) and body weight (**C**) were measured at the indicated time. At the end of the experiment, the mice were sacrificed, and the tumor tissues were dissected and weighed (**B**). The data are expressed as the mean ± SD (8–9 mice per group). NS, not significant; ^a^ *p* < 0.05; ^b^ *p* < 0.05; ^c^ *p* < 0.001, difference with the control group (0 mg/kg of artesunate). (**D**) C.B.17SC scid^−/−^ female mice (5–6 weeks old) bearing Rh65 xenografts were treated i.p. with DHA (100 mg/kg). After the treatment for the indicated time, the mice were sacrificed, and the tumor tissues were collected and frozen in liquid N_2_. Western blotting was performed with indicated antibodies. Control means non-treatment with DHA; T1, T2, and T3 represent tumors #1, #2, and #3, respectively.

Artesunate, a pro-drug of DHA, has been in clinical trials for treatments of lung, colon, breast, and cervical cancer in adults [19]. To assess the potential of DHA for treatments of RMS, we evaluated the anticancer activity of artesunate in Rh30 xenografts in SCID mice. The results showed that treatments with artesunate (i.p. once daily at 25, 50, 100, and 150 mg/kg body weight) for 32 days in a dose-dependent manner inhibited the tumor growth (volume) of Rh30 xenografts in mice, by 23.6, 50.8%, 70.7%, and 80.3%, respectively, compared to the vehicle treatment (Figure 9A; Supplementary Figure S4). Artesunate treatments displayed similar inhibitory effects on the tumor weight (Figure 9B). Of note, no obvious toxicity was observed in all the treated groups except for the 150 mg/kg group, in which the average body weight of mice decreased slightly but not significantly (Figure 9C).

To study the in vivo effects of DHA on AMPK and mTORC1, SCID mice bearing with Rh65 xenografts were treated i.p. with DHA (100 mg/kg body weight). At 2, 4, 8, and 24 h of post-treatment, the mice were sacrificed, and the tumor tissues were collected. Our Western blotting analysis revealed that treatment with DHA for 8 h remarkably inhibited p-S6 (S235/236) and p-4E-BP1 (T70) but did not apparently affect p-Akt (S473) in the tumors (Figure 9D), in line with our in vitro results (Figure 1B). Interestingly, DHA treatment also time-dependently induced p-AMPK (T172) in vivo (Figure 9D), also consistent with the in vitro data (Figure 7). The results underline that artesunate or DHA has a great potential for RMS therapy.

## 4. Discussion

Increasing evidence suggests that DHA exerts its anticancer activity primarily by inhibiting mTORC1 signaling in tumor cells [20,21,22,23,24]. However, how DHA inhibits mTORC1 is unknown. mTOR can be inhibited due to the binding of compounds (either allosterically by rapalogs or directly by mTOR kinase inhibitors, e.g., INK128 and AZD8055) [5,6]. In addition, mTOR can be inhibited indirectly via multiple mechanisms, including the inhibition of the IGF-1R-PI3K-Akt and Ras-Raf-MEK-Erk pathways and the activation of the HIF1-REDD1 and AMPK pathways [5,6]. Recently, Du et al. has shown that DHA induces autophagy of leukemia cells partly by inhibiting p-mTOR, p-S6K1, and p-S6 and activating p-AMPK in leukemia (HL60 and THP-1) cells [26], but whether DHA-induced mTORC1 inhibition is a consequence of AMPK activation has not been resolved. Here, for the first time, we present evidence that DHA inhibits mTORC1 neither by directly binding to mTOR or FKBP12 nor by indirectly inhibiting the IGF-1R-PI3K-Akt and Erk pathways or activating PTEN, HIF-1α, and REDD1. Instead, DHA inhibits mTORC1 by activating the AMPK pathway in tumor cells. Furthermore, DHA is able to induce the dissociation of raptor from mTOR and inhibit the activity of mTORC1.

Current chemotherapeutic treatments for pediatric RMS may cause long-term side effects, such as secondary cancers and infertility [1,3]. Artemisinins have been widely used for treatment of malaria in children and adults for decades, and their safety is clinically proven [16,17,18]. Especially, artesunate, a pro-drug of DHA, has been in clinical trials for treatment of lung, colon, breast, and cervical cancer in adults [19]. Therefore, we explored whether DHA can be repositioned for treatment of RMS. Here, our in vitro and in vivo data indicate that DHA is able to potently inhibit the growth of RMS by inhibiting mTORC1 signaling. Our findings support that DHA has a great potential for treatment of RMS. 

Here, we found that DHA preferably targets RMS tumor cells, but not normal cells (Figure 1A). The possible reasons are discussed here. One the one hand, it has been documented that the *PAX3/7*–*FOXO1* fusion in RMS tumor cells results in constitutively active mTOR signaling [1,2,3]. RMS cells, unlike normal cells (normal HSMCs), mouse skeletal muscle cells (C2C12), and normal HDFs, had hyperactive mTOR signaling (see Figure 1C), so they are apparently addictive to mTOR signaling for growth, proliferation, and survival. This may be one of the major reasons why DHA preferably targets RMS cells but not normal cells. On the other hand, as shown in Figure 6, in response to DHA, AMPK can be activated in RMS cells, but not in normal cells. This may be partly related to the differential effects of DHA on the cellular levels of ATP in RMS cells and normal cells. It is well known that AMPK can be activated in response to energy stress [5,6]. In our study, we observed that treatment with DHA for 18 h reduced the cellular ATP level in Rh30 cells (Supplementary Figure S5C). Of note, the 24 h treatment with 30 μM of DHA reduced the intracellular ATP level by 80% (compared to the control) in tumor cells (Rh30), whereas the same treatment only reduced the ATP level by ~30% in normal cells (C2C12) (Supplementary Figure S5D), in line with tumor-selective effects of DHA on cell proliferation/viability and mTORC1. At this stage, we could not rule out other possibilities, such as differential expression levels of drug efflux transporters (e.g., P-glycoprotein and multidrug resistance proteins).

In this study, we noticed that the treatment with artesunate dose-dependently inhibited the tumor growth of Rh30 xenografts in mice. The treatment with artesunate (50 and 100 mg/kg) potently inhibited the tumor growth (by 58% and 65%, respectively; *p* < 0.01), but had no marked effect on the body weight of the animals, reinforcing the good safety of artesunate. However, we have to acknowledge that single treatment with artesunate, even at 150 mg/kg, failed to result in tumor regression (Figure 9), implying that combination treatments are necessary. Given that the standard chemotherapy for RMS is the combination of vincristine, actinomycin D, and cyclophosphamide [1,3], it is worthy to further study whether artesunate synergizes with these chemotherapeutic agents in RMS.

Here, we found that DHA (0−30 μM) did not influence the phosphorylation of Akt (S473) in RMS (Rh18, Rh28, Rh36, Rh30, Rh41, and RD) cells (Figure 1), which is consistent with our previous observation in Ewing sarcoma (Rh1) cells [21]. Rapamycin has been shown to inhibit mTORC1 but induces p-Akt (S473) in RMS cells [40]. These findings suggest that the effect of DHA on p-Akt is different from that of rapamycin. It is known that rapalogs inhibit mTORC1 but activate Akt through the S6K1-IRS1 negative feedback mechanism [12,13,14]. Rapalogs-activated Akt is regarded as a major drawback contributing to their mild anticancer activity in most clinical settings, as activated Akt can promote cancer cell survival [5,6]. A number of clinical trials of artesunate are ongoing for treatments of various cancers [19]. Since rapalogs alone lack efficacy in treatments of most types of cancer, including RMS [5,6,10,11], it would be interesting to determine whether DHA or artesunate, as an anti-cancer agent, is clinically superior to rapalogs.

Of note, a recent report has shown that a treatment with 40 μM of DHA reduces the protein levels of HIF-1α and p-Akt (S473) in prostate LNCaP cells [30]. This is in contrast to our results that a treatment with 0.3–30 μM of DHA did not alter the levels of HIF-1α (Figure 5A) and p-Akt in both Rh30 (Figure 1B) and Rh1 cells [21]. Whether the discrepancy is due to different concentrations of DHA used remains to be determined. We have noticed that curcumin at high concentrations (>20 μM), which is clinically irrelevant, is able to inhibit both mTORC1 and mTORC2 [41].

It has been described that AMPK activation induces phosphorylation of p53 on serine 15 [50] and p53 activation can inhibit mTORC1 [51]. In the present study, we found that DHA was able to inhibit mTORC1 in multiple cell lines, of which Rh30, RD and Rh1 cells expressed mutant p53 alleles (Rh30 Arg273→Cys; RD Arg248→Trp; Rh1: Tyr220→Cys), losing the function of p53 [21]. Thus, our results suggest that the AMPK-p53 pathway is dispensable for DHA-induced mTORC1 inhibition. Since activated AMPK can also inhibit mTORC1 by activating the formation of TSC1/2 complex and/or phosphorylating raptor (S792) [8,9], to better understand how DHA inhibits mTORC1, further research is required to define whether the AMPK-TSC and AMPK-raptor pathways are involved in DHA-induced mTORC1 inhibition.

A new question is that how DHA activates AMPK. Our molecular docking indicates that although DHA has the possibility to bind the interface cavity of the carbohydrate-binding module (CBM, also known as the glycogen-binding domain) and the kinase domain of AMPK, the interactions of DHA with the protein complex are much weaker than those of A-769662 (a known AMPK activator) [46], consistent with the calculated scoring function (GOLD scores: 44.73 for DHA vs. 75.77 for A-769662). Further research is needed to confirm whether DHA is able to bind to α, β, or γ subunit of AMPK or not. 

AMPKα (T172) can be activated by liver kinase B1 (LKB1) in response to low levels of energy (ATP), by transforming growth factor β-activated kinase 1 (TAK1) due to increased cytokines and/or by calmodulin-dependent kinase kinase β (CaMKKβ) upon elevated intracellular Ca^2+^ levels [52,53,54]. Besides, in response to various stimuli (e.g., oxidative stress, glucose, tumor necrosis factor-α, and palmitate), AMPKα (T172) can be dephosphorylated and inactivated by protein phosphatase 1 (PP1) [55], protein phosphatase 2A (PP2A) [56,57,58], protein phosphatase 2B (PP2B, also calcineurin) [59], protein phosphatase 2C (PP2C) [58], and protein phosphatase 5 (PP5) [60]. In our study, we noticed that the treatment with DHA (0–30 μM) for 24 h did not alter intracellular Ca^2+^ levels in Rh30 and RD cells. Of interest, 8 h or 24 h treatments with DHA (0−30 µM) induced ROS in Rh30 cells in a dose-dependent manner (Supplementary Figure S5A,B). In addition, the treatment with DHA (10 or 30 µM) for 18–24 h significantly reduced the cellular ATP levels in Rh30 and RD cells (Supplementary Figure S5C,D). Therefore, it would be interesting to figure out whether DHA-induced activation of AMPK is mediated by any of these kinases and/or phosphatases.

In the present study, we also observed that DHA induced the dissociation of raptor from mTOR (Figure 8A). AMPK-mediated phosphorylation of both TSCs and raptor does not cause disassembly of mTORC1 [5,6]. It has been shown that GRp58/ERp57 is involved in the assembly of mTORC1 and positively regulates mTORC1 signaling at the cytosol and the cytosolic side of the ER [61]. Further research is needed to address whether DHA disrupts mTORC1 by targeting GRp58/ERp57.

## 5. Conclusions

**Figure 10:**
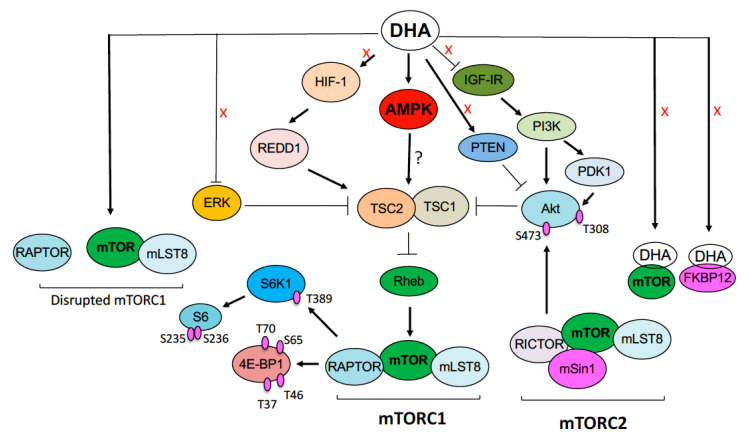
A proposed model of mTORC1 inhibition by DHA. DHA does not directly bind to mTOR or FKBP12. Besides, DHA alters neither the phosphorylation of IGF-IR/PI3K/Akt/PTEN and Erk1/2, nor the expression of HIF1α/REDD1 in tumor cells. Instead, DHA inhibits mTORC1 by activating the AMPK pathway. In addition, DHA induces the dissociation of raptor from mTOR and inhibit the activity of mTORC1. Abbreviations: 4E-BP1, eukaryotic initiation factor 4E (eIF4E) binding protein 1; Akt, protein kinase B; AMPK, AMP-activated protein kinase; DHA, dihydroartemisinin; ERK, extracellular signal-regulated kinases; FKBP12, FK506 binding protein 12; HIF1, hypoxia-inducible factor 1; IGF-1R, insulin-like growth factor-1 receptor; mLST8, mammalian lethal with sec-13 protein 8; mSin1, mammalian stress-activated protein kinase-interacting protein 1; mTOR, mammalian target of rapamycin; mTORC1, mTOR complex 1; mTORC2, mTOR complex 2; PDK1, PI3K, phosphoinositide 3-kinase; PTEN, phosphatase and tensin homolog; RAPTOR, regulatory-associated protein of mTOR; REDD1, Rheb, Ras homolog enriched in brain; RICTOR, rapamycin-insensitive companion of mTOR; S, serine; S6, ribosomal protein S6; S6K1, p70 S6 kinase 1; T, threonine; TSC1, tuberous sclerosis complex 1; TSC2, tuberous sclerosis complex 2.

Here, we showed that DHA inhibited mTORC1 in tumor cells not through direct binding to mTOR or FKBP12, but via indirect mechanisms. Apparently, DHA altered neither the phosphorylation of IGF-IR/PI3K/Akt/PTEN and Erk1/2, nor the expression of HIF1α/REDD1 in tumor cells. Instead, DHA inhibited mTORC1 by activating the AMPK pathway (Figure 10). Additionally, DHA was able to induce dissociation of raptor from mTOR and inhibit the activity of mTORC1. To our knowledge, this is the first study to unveil how DHA inhibits mTORC1 in tumor cells. In addition, our in vitro and in vivo data demonstrate that DHA has a great potential for RMS treatment. To facilitate repurposing this anti-malaria agent for RMS treatment, further research is warranted to determine whether artesunate (alone or in combination with other anticancer agents) is more effective than rapalogs in mouse tumor models.

## Figures and Tables

**Table 1:** Scoring function values of dihydroartemisinin and rapamycin in molecular docking studies, in which the agents were docked into the active pocket of FKBP12 (PDB ID: 3FAP).

Compound	LibDock Score	GOLD Score
Rapamycin	101.40	148.24
Dihydroartemisinin	29.72	92.98

## Data Availability

All the data presented in this study are included in this article.

## Supplementary Materials

The following are available online at https://www.mdpi.com/article/10.3390/cells10061363/s1, Figure S1: Knockdown of raptor or rictor inhibits RMS cell growth. Figure S2: Inhibition of AMPK attenuates DHA-induced inhibition of mTORC1 in Rh30 cells. Figure S3: Ectopic expression of kinase-dead AMPK attenuates DHA-induced inhibition of mTORC1 in Rh30 cells. Figure S4: Artesunate inhibits the tumor growth of Rh30 xenografts in SCID mice. Figure S5: DHA increases cellular ROS and reduces ATP levels in tumor cells.
