# Supplementary material for: Dihydroartemisinin Inhibits mTORC1 Signaling by Activating the AMPK Pathway in Rhabdomyosarcoma Tumor Cells
Source: Cells. 2021 Jun 1;10(6):1363. doi: 10.3390/cells10061363 (PMC8226784; doi:10.3390/cells10061363)
Supplement: Supplementary file 1 [file cells-10-01363-s001.zip › cells-1170695-supplementary.pdf]

cells MDPI

Supplementary Material

Dihydroartemisinin Inhibits mTORC1 Signaling by Activating the AMPK Pathway in Rhabdomyosarcoma Tumor Cells

Jun Luo [1,2,†], Yoshinobu Odaka [1,†,‡], Zhu Huang [1,3,†], Bing Cheng [1], Wang Liu [1], Lin Li [1], Chaowei Shang [1], Chao Zhang [1,4,5], Yang Wu [1,6], Yan Luo [1,6], Shengyong Yang [6], Peter J. Houghton [7], Xiaofeng Guo [2,*] and Shile Huang [1,8,*]

[1] Department of Biochemistry and Molecular Biology, Louisiana State University Health Sciences Center, Shreveport, LA 71130-3932, USA; junluo@scau.edu.cn (J.L.); odakayu@ucmail.uc.edu (Y.O.); huangzhu@xmu.edu.cn (Z.H.); bing.cheng@lsuhs.edu (B.C.); wliu6@kumc.edu (W.L.); lin.li@lsuhs.edu (L.L.); chaowei.shang@lsuhs.edu (C.S.); zhangchao@ahmu.edu.cn (C.Z.); wuyang@scu.edu.cn (Y.W.); yan.luo@mayo.edu (Y.L.)
[2] College of Veterinary Medicine, South China Agricultural University, Guangzhou 510642, China
[3] Research Center of Aquatic Organism Conservation and Water Ecosystem Restoration in Anhui Province, Anqing Normal University, Anqing 246011, China
[4] Key Laboratory of National Health and Family Planning Commission on Parasitic Disease Control and Prevention, Jiangsu Institute of Parasitic Diseases, Wuxi 214064, China
[5] Jiangsu Provincial Key Laboratory on Parasite and Vector Control Technology, Jiangsu Institute of Parasitic Diseases, Wuxi 214064, China
[6] State Key Laboratory of Biotherapy and Cancer Center, West China Hospital, Sichuan University, Chengdu 610041, China; yangsy@scu.edu.cn
[7] Greehey Children's Cancer Research Institute, University of Texas Health Science Center, San Antonio, TX 78229-3000, USA; houghtonp@uthscsa.edu
[8] Department of Hematology and Oncology, Louisiana State University Health Sciences Center, Shreveport, LA 71130-3932, USA
* Correspondence: xfguo@scau.edu.cn (X.G.); shile.huang@lsuhs.edu (S.H.); Tel: +86-20-38295980 (X.G.); +1-318-675-7759 (S.H.)
† These authors contributed equally to this work.
‡ Current address: Biology Department, University of Cincinnati Blue Ash College, Blue Ash, OH 45236, USA

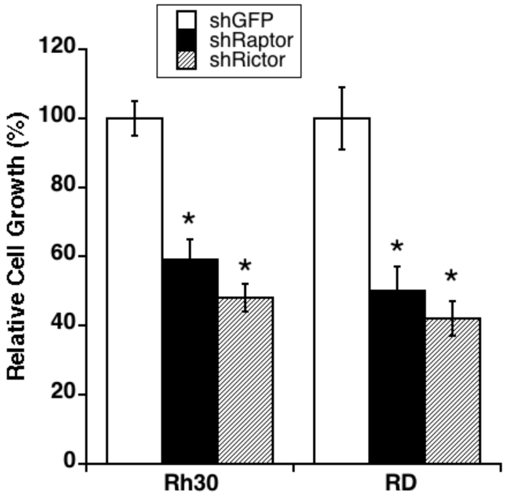

Figure S1. Knockdown of raptor or rictor inhibits RMS cell growth. Rh30 and RD cells, infected with lentiviral shRNA to raptor, rictor, or GFP (control), were seeded in 6-well plates (all at 4×10^4 cells/well) and grown for 72 h, followed by cell counting using a Beckmann Coulter counter. Shown are mean values ± SD ($n = 3$). $^{*}p < 0.05$, difference versus the control group (sh-GFP).

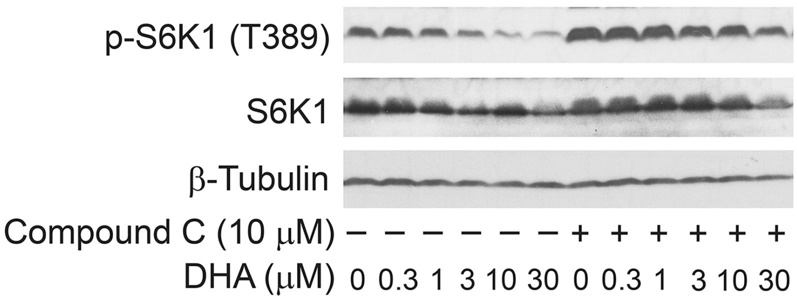

Figure S2. Inhibition of AMPK attenuates DHA-induced inhibition of mTORC1 in Rh30 cells. Rh30 cells were pretreated with Compound C (10 μM) for 2 h, and then exposed to DHA (0–30 μM) for 24 h. The cell lysates were subject to Western blotting with indicated antibodies.

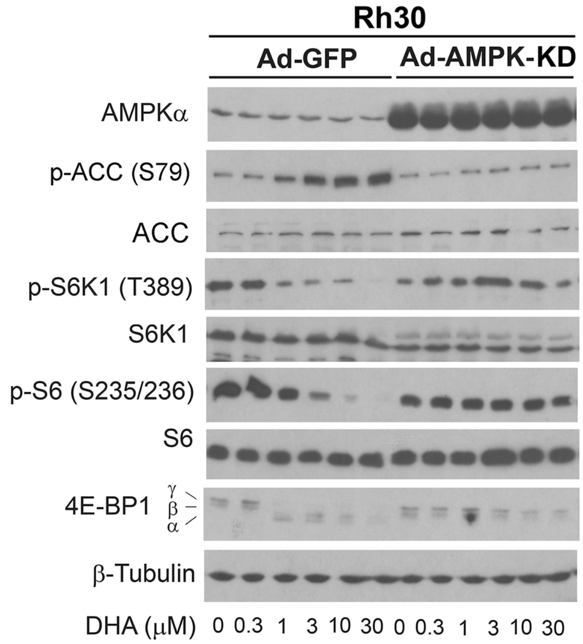

Figure S3. Ectopic expression of kinase-dead AMPK attenuates DHA-induced inhibition of mTORC1 in Rh30 cells. Rh30 cells were infected with recombinant adenovirus expressing myc-tagged kinase-dead AMPK (Ad-AMPK-KD) or GFP (Ad-GFP) for 24 h, and then treated with DHA (0-30 μM) for another 24 h, followed by Western blotting with indicated antibodies.

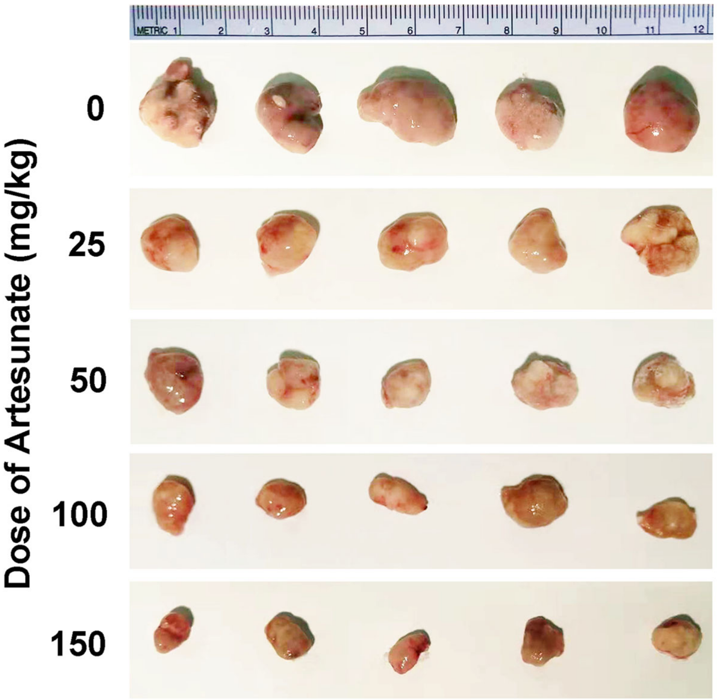

Figure S4. Artesunate inhibits the tumor growth of Rh30 xenografts in SCID mice. C.B.17SC *scid*$^{-/-}$ female mice (5~6 weeks old) bearing Rh30 xenografts were treated (i.p.) with artesunate at the indicated doses or vehicle (0.9%NaCl) once daily. At the end (Day 33) of the experiment, the mice were sacrificed and the tumor tissues were dissected. Shown are the representative images of isolated tumors.

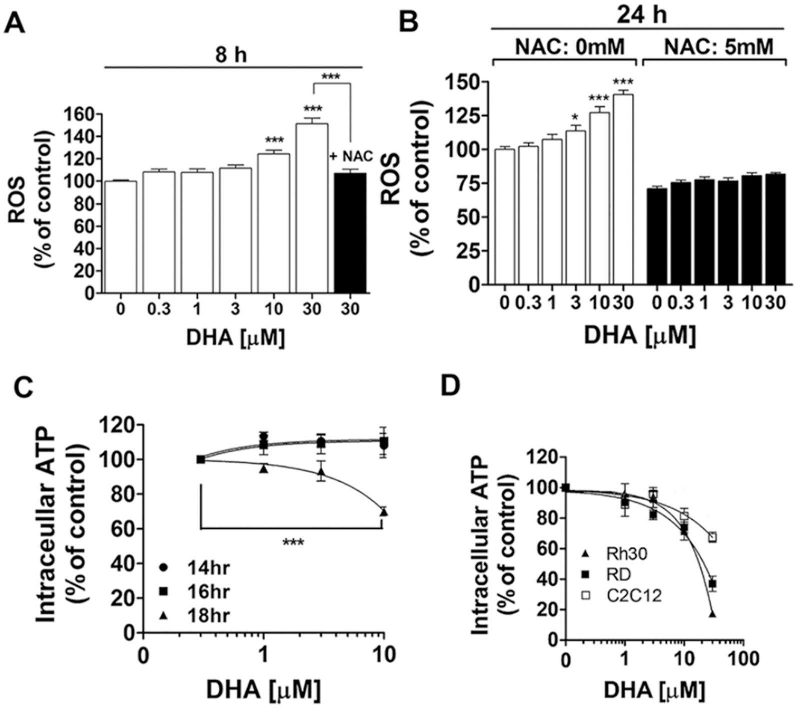

Figure S5. DHA increases cellular ROS and reduces ATP levels in tumor cells. (**A**,**B**) Rh30 cells were preloaded with CM-H$_2$DCFDA (10 μM) for 30 min, followed by treatment with DHA (0-30 μM) in

the presence or absence of 5 mM NAC (N-acetyl cysteine) for 8 h (**A**) or 24 h (**B**), before ROS detection. (**C**) Rh30 cells were treated with DHA (0–10 μM) for 14-18 h, (**D**) indicated cells were treated with DHA (0-30 μM) for 24 h, followed by ATP assay using ATPlite™ Luminescence Assay System (PerkinElmer). Results are means ± SE (n = 6). *$p < 0.05$, ***$p < 0.001$.
